# Combined Laser Therapy for Post-sclerotherapy Hyperpigmentation Following Nicolau Syndrome: A Case Report

**DOI:** 10.7759/cureus.82375

**Published:** 2025-04-16

**Authors:** Nikolai Alekseev, Valeriia Mishanina

**Affiliations:** 1 Laser Medical Physics, Independent Researcher, Irvine, USA; 2 Dermatology, Educational Center of the Project Laboratory of Laser Technologies, Moscow, RUS

**Keywords:** er:yag, ipl, laser therapy, nicolau syndrome, post-sclerotherapy hyperpigmentation, q-switched nd:yag

## Abstract

Post-sclerotherapy hyperpigmentation is a common adverse effect, particularly when complicated by Nicolau syndrome, a rare injection-related event that can lead to tissue necrosis and persistent pigmentary changes. We report a case of a 40-year-old woman with Fitzpatrick skin type II, who developed longstanding hyperpigmentation in the right popliteal area following polidocanol sclerotherapy. Clinical and dermoscopic examination revealed mixed melanin and hemosiderin deposition. The patient was successfully treated using a quadrant-specific multimodal laser approach combining intense pulsed light (IPL), Q-switched neodymium-doped yttrium aluminum garnet (Nd:YAG), and erbium-doped yttrium aluminum garnet (Er:YAG) lasers over two sessions. Notable pigment clearance, full crust exfoliation, and restoration of normal skin tone were achieved without adverse effects. Independent expert evaluation and patient-reported outcomes confirmed the treatment’s efficacy and high tolerability.

## Introduction

Sclerotherapy is a widely utilized, minimally invasive technique for the treatment of various venous disorders, including telangiectasias, reticular veins, and larger varicose veins, owing to its effectiveness, affordability, and ease of application. Despite its benefits, post-procedural complications such as hyperpigmentation remain a common concern. A systematic review by Bossart et al. reported hyperpigmentation rates ranging from 7.4% to 32.5% following polidocanol-based sclerotherapy for telangiectasias and reticular veins, with persistent pigmentation beyond one year observed in up to 17.5% of cases treated with foam sclerosants [1].

Nicolau syndrome, also known as embolia cutis medicamentosa, is a rare but serious complication of injections, including sclerotherapy. It is characterized by acute pain, livedoid dermatitis, tissue ischemia, and possible necrosis, often followed by post-inflammatory hyperpigmentation and scarring [2,3]. Although the exact pathogenesis remains unclear, mechanisms such as arterial embolism, vasospasm, and endothelial injury have been implicated [4].

The management of hyperpigmentation following Nicolau syndrome poses a therapeutic challenge due to the involvement of both melanin and hemosiderin, alongside dermal remodeling and inflammation [5]. Conventional topical treatments often yield unsatisfactory results, prompting the use of laser-based modalities [6]. Intense pulsed light (IPL), Q-switched neodymium-doped yttrium aluminum garnet (Nd:YAG) laser, and erbium-doped yttrium aluminum garnet (Er:YAG) laser are widely used in treating pigmentation disorders. These modalities were selected in this case for their complementary mechanisms: IPL targets melanin chromophores in the epidermis, Q-switched Nd:YAG effectively addresses deeper pigmented particles, including hemosiderin, and Er:YAG offers fractional resurfacing to support dermal remodeling.

This case report describes the successful treatment of persistent hyperpigmentation secondary to Nicolau syndrome using a multimodal laser approach, illustrating the potential of combining IPL, Q-switched Nd:YAG, and Er:YAG lasers in managing complex pigmentary complications.

## Case presentation

A 40-year-old female patient with Fitzpatrick skin type II presented to our dermatology department for evaluation of persistent hyperpigmentation localized to the right popliteal region following sclerotherapy performed for varicose veins. At the time of consultation, the lesion had been present for approximately 10 months, significantly impacting the patient's quality of life and causing notable psychological distress.

**Figure 1:**
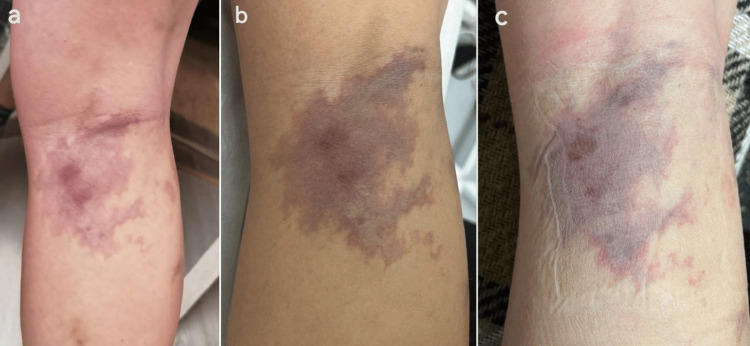
Clinical evolution of the lesion in the early post-sclerotherapy period. The initial presentation (a) shows a violaceous discoloration with irregular borders localized to the right popliteal region, consistent with post-injection vascular injury. During clinical evaluation (b), topical treatment was initiated without complications. Follow-up examination (c) revealed early signs of improvement, with a visible reduction in pigmentation and lesion size.

The patient's medical history revealed that she underwent two sessions of liquid sclerotherapy utilizing polidocanol (fibrovein 3% diluted in 40% glucose solution at a concentration of 1/10). The procedures were performed using the cryosclerotherapy technique under ultrasound guidance, with injections administered on November 14, 2022 (11 mL solution) and December 19, 2022 (16 mL solution), targeting reticular veins. Immediately following the second session, the patient experienced acute severe pain accompanied by localized skin blanching, clinical features suggestive of Nicolau syndrome. Subsequently, skin necrosis developed at the injection site, eventually healing and evolving into persistent hyperpigmentation (Figure 1).

**Figure 2:**
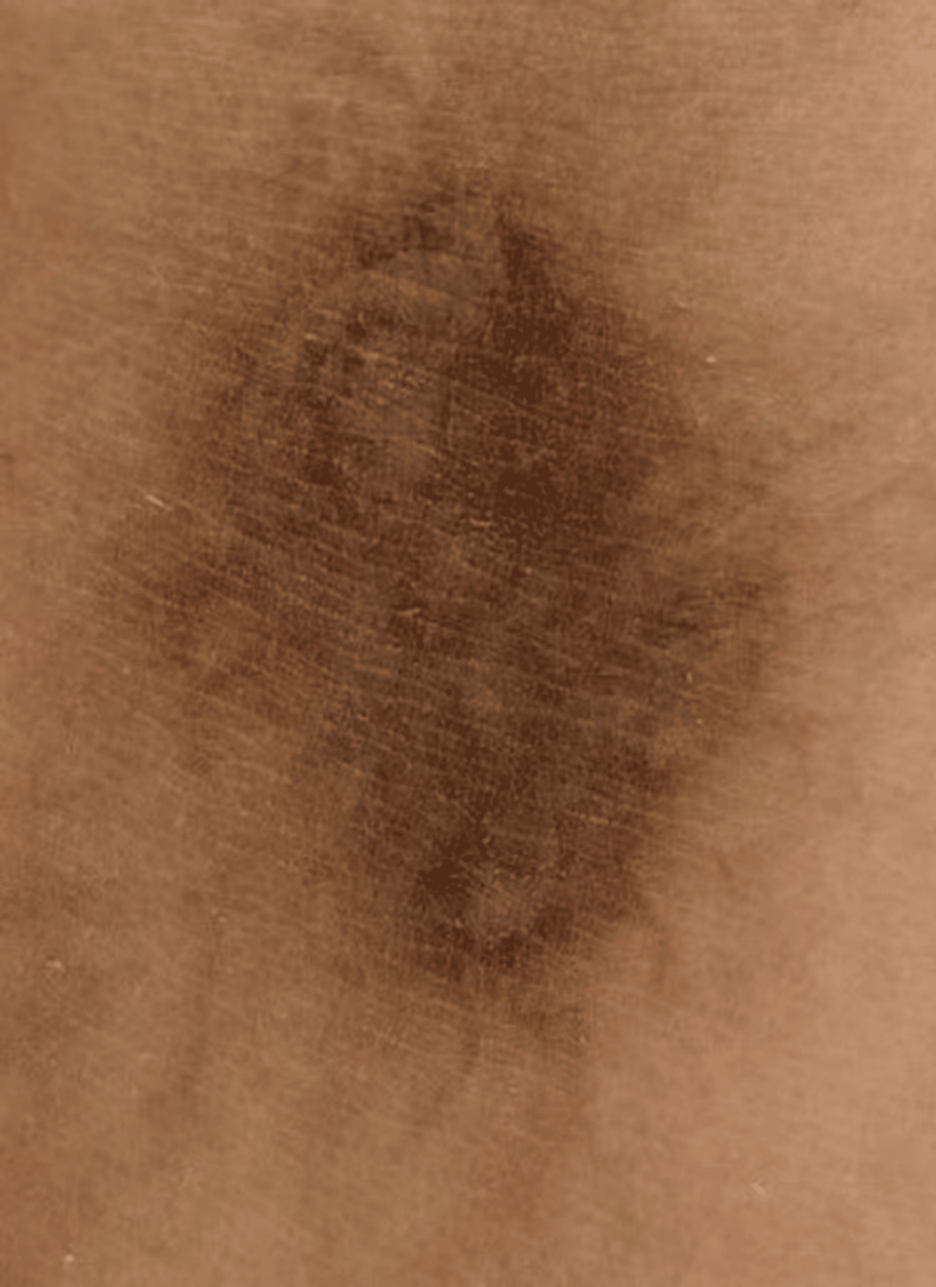
Clinical appearance of the lesion before laser treatment, showing irregular, heterogeneous hyperpigmentation. Dermoscopy revealed superficial and deep pigment deposits consistent with melanin and hemosiderin.

Clinical examination at presentation revealed a hyperpigmented lesion measuring approximately 4 × 6 cm, exhibiting irregular borders and uneven pigment distribution. The affected skin demonstrated notable thickening and post-inflammatory changes. Dermatoscopic evaluation confirmed the coexistence of superficial and deep pigment deposits, indicative of melanin and hemosiderin accumulation (Figure 2).

Given the patient's history and characteristic clinical presentation, a diagnosis of post-sclerotherapy hyperpigmentation secondary to Nicolau syndrome was established. After detailed counseling, a multimodal laser treatment approach was planned and executed utilizing a combination of IPL, Q-switched Nd:YAG, and Er:YAG lasers.

**Figure 3:**
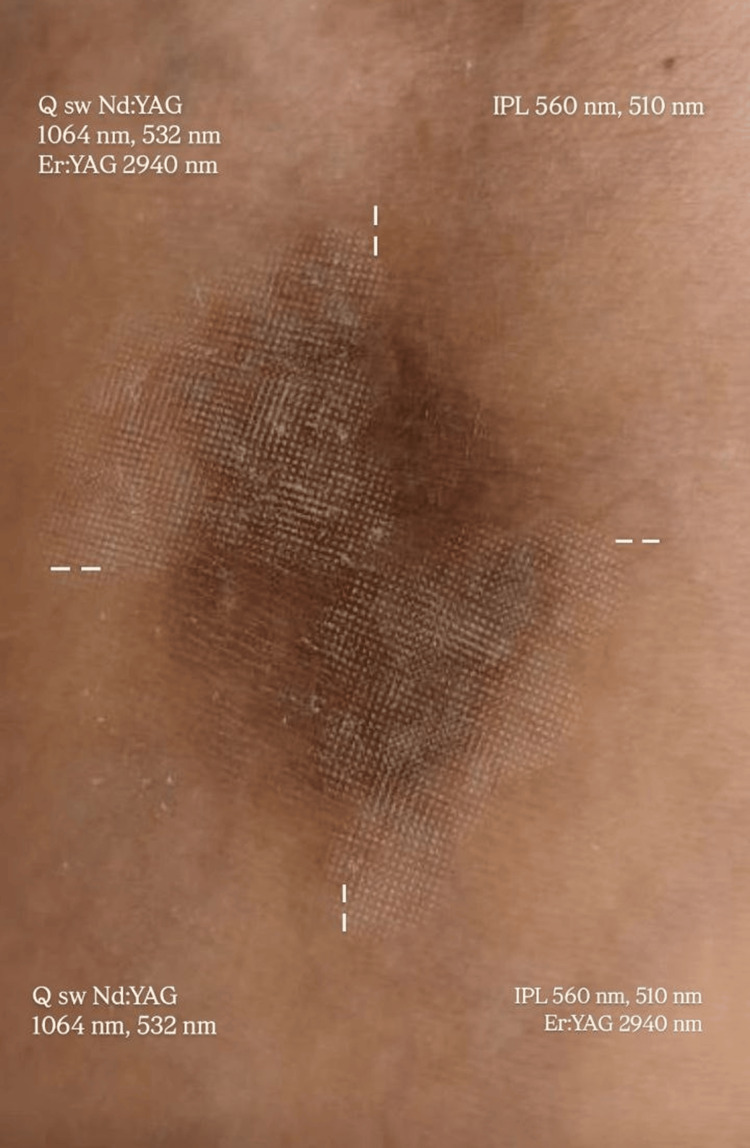
Quadrant-specific laser treatment approach showing distribution of IPL, Q-switched Nd:YAG, and Er:YAG modalities across the lesion. IPL: intense pulsed light; Nd:YAG: neodymium-doped yttrium aluminum garnet laser; Er:YAG: erbium-doped yttrium aluminum garnet laser.

The treatment protocol consisted of two sessions conducted eight weeks apart, with each session addressing all four quadrants of the affected area. Cooling systems integrated into the laser devices ensured that procedures were painless, and no adverse effects were reported. Quadrant-specific protocols included IPL (560 nm, 6 ms, 16 J/cm² and 510 nm, 7 ms, 17 J/cm²) for the upper right quadrant, combined IPL (560 nm, 6 ms, 16 J/cm²) and Er:YAG (2940 nm, 0.9 J, fractional setting: 100 micro-thermal zones) for the lower right quadrant, combined Q-switched Nd:YAG (1064 nm, 8 ns, 2 J/cm²; 532 nm, 8 ns, 1 J/cm²) and Er:YAG (2940 nm, 0.9 J, fractional setting: 100 micro-thermal zones) for the upper left quadrant, and Q-switched Nd:YAG alone (1064 nm, 8 ns, 2 J/cm²; 532 nm, 8 ns, 1 J/cm²) for the lower left quadrant (Table 1 and Figure 3).

**Table 1:** Quadrant-specific laser treatment protocol. IPL: intense pulsed light; Nd:YAG: neodymium-doped yttrium aluminum garnet laser; Er:YAG: erbium-doped yttrium aluminum garnet laser; nm: nanometers; ms: milliseconds; ns: nanoseconds; J/cm²: Joules per square centimeter.

Quadrant	Treatment method	Parameters
Upper right	IPL	1 pass 560 nm/6 ms/16 J/cm²; 1 pass 510 nm/7 ms/17 J/cm²
Lower right	IPL + Er:YAG	IPL: 1 pass 560 nm/6 ms /16 J/cm²; Er:YAG: 2940 nm, 0.9 J, fractional 100 (micro-thermal zones)
Upper left	Q-switched Nd:YAG + Er:YAG	Nd:YAG-Qsw: 1 pass 1064 nm/ 8 ns/2 J/cm²; 1 pass 532 nm/8 ns/1 J/cm²; Er:YAG: 2940 nm, 0.9 J, fractional 100 (micro-thermal zones)
Lower left	Q-switched Nd:YAG	Nd:YAG-Qsw: 1 pass 1064 nm/8 ns/2 J/cm²; 1 pass 532 nm/8 ns/1 J/cm²

**Figure 4:**
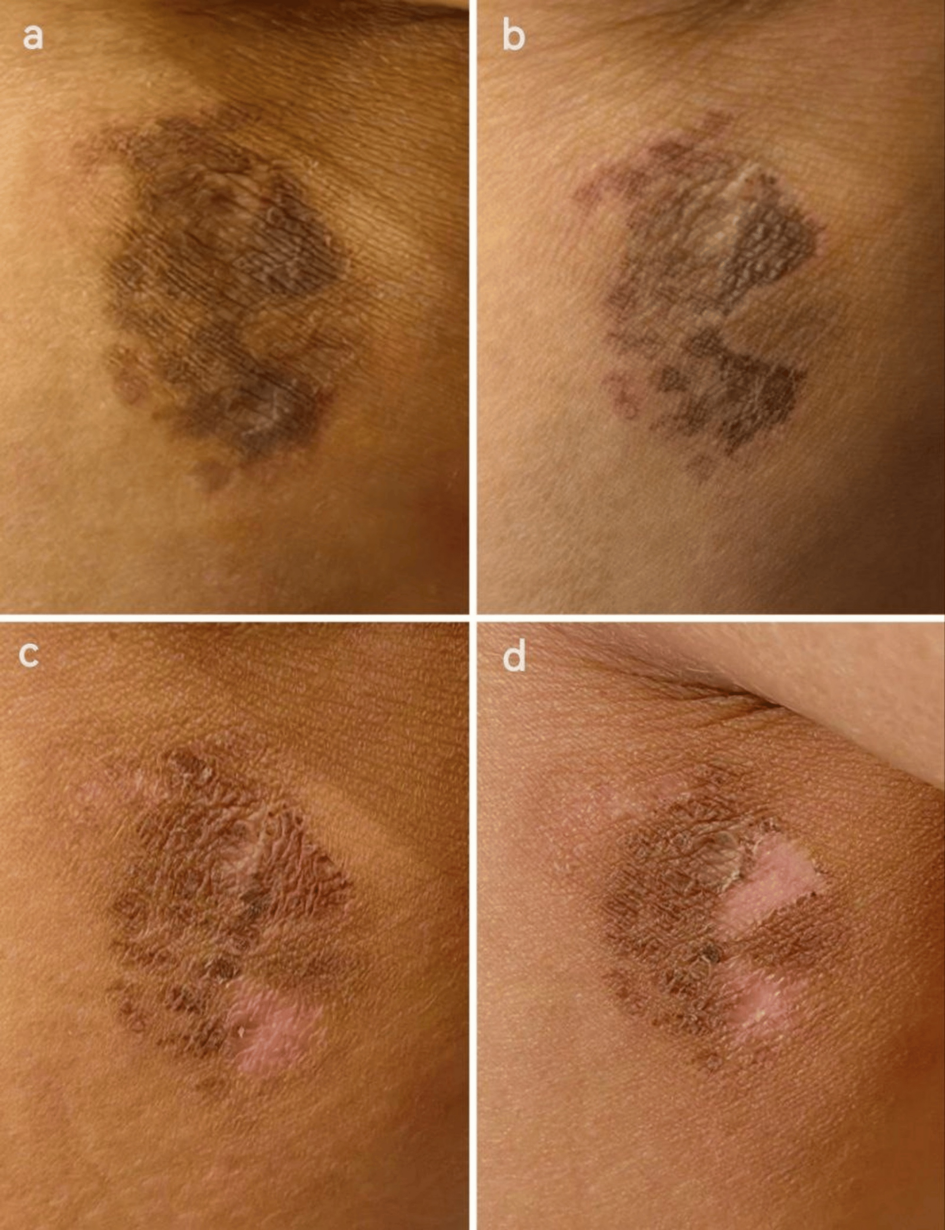
Evolution of the lesion following the first laser session. (a) Initial darkening observed across all quadrants, with partial sparing in the upper-right quadrant due to uneven stencil application. (b) Increased pigmentation with thin crust formation, particularly in IPL-treated areas. (c) Marked lightening in the lower-right quadrant following exfoliation. (d) Partial pigment clearance in the upper-right quadrant. IPL: intense pulsed light.

**Figure 5:**
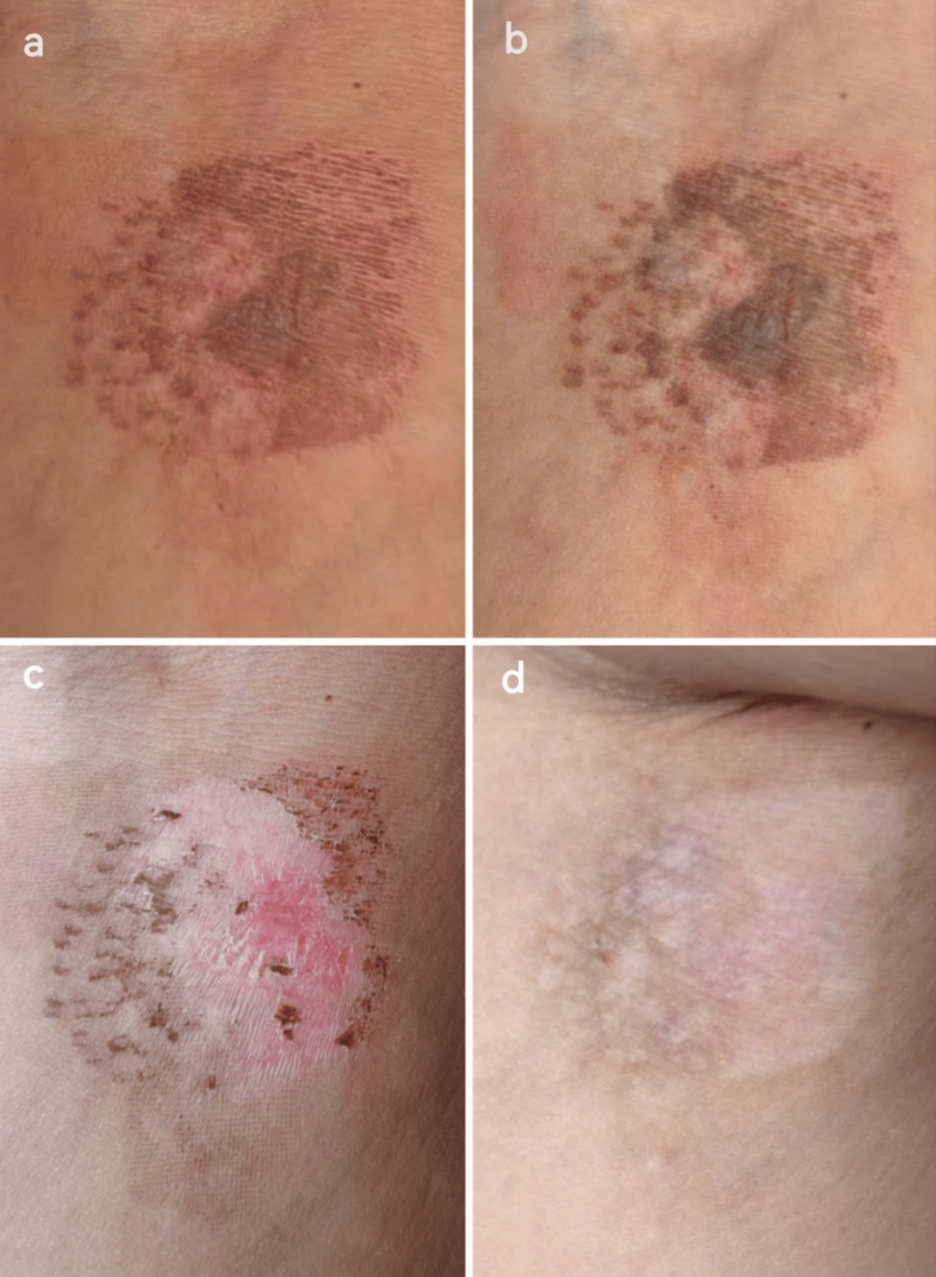
Clinical evolution following the second laser session. (a) Immediate post-procedure appearance with mild erythema and laser grid marks. (b) Progressive pigment breakdown noted across all quadrants. (c) Pronounced exfoliation and significant lightening observed. (d) Marked pigment reduction and near-complete clearing of the lesion.

Pigment clearance was evaluated independently using a standardized visual scale (0% = no improvement, 100% = full clearance), and patient satisfaction was assessed using the visual analog scale (VAS; 0 = no improvement, 10 = excellent improvement). Both evaluations confirmed significant pigmentation reduction in all quadrants, with the IPL and Er:YAG combination achieving complete resolution and Q-switched Nd:YAG monotherapy showing moderate improvement (Figures 4, 5).

Independent expert evaluation assessed parameters, including skin color and hyperpigmentation, contrast reduction, degree of hypopigmentation, and overall clinical efficacy. Scores notably improved from the first to the second session, ultimately achieving an overall clinical efficacy score of 8 out of 10, reflecting high efficacy in hyperpigmentation reduction and improved skin texture (Table 2).

**Table 2:** Independent expert evaluation results.

Parameter	After the first session	After the second session	Expert comments
Skin color and hyperpigmentation	6.5	9.5	Significant lightening, especially notable in the left quadrants after the second session
Contrast reduction with the surrounding tissue	5.5	9.0	Marked reduction in contrast after the second session
Degree of hypopigmentation	3.0	5.5	Mild hypopigmentation observed after the second session
Overall clinical efficacy (weighted score)	5.0	8.0	Second session exceeded expectations; high overall efficacy

**Figure 6:**
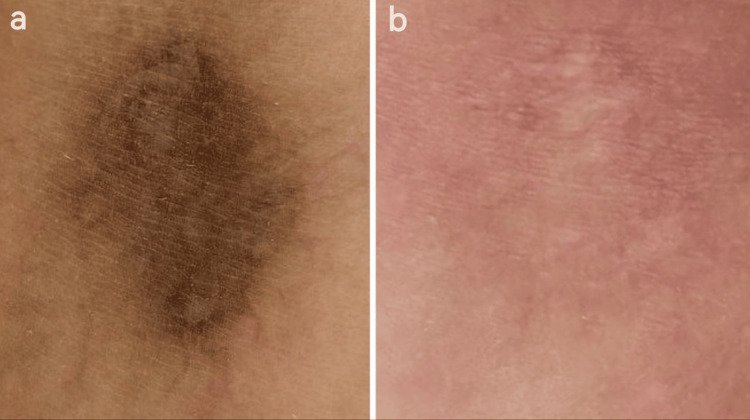
Pre- and post-treatment comparison. (a) Baseline hyperpigmentation. (b) Near-complete pigment clearance after treatment.

Patient satisfaction, measured by the Patient Satisfaction Scale (PSS; 1 = completely unsatisfied to 5 = completely satisfied), significantly increased from an average score of 3.8 after the first procedure to 4.7 following the second. The patient reported significant improvement in skin appearance, minimal procedural discomfort, and expressed particular satisfaction with the near-total resolution of hyperpigmentation (Table 3 and Figure 6).

**Table 3:** Patient satisfaction results.

Treatment stage	Average Patient Satisfaction Scale (PSS) score	Key patient comments
After the first procedure	3.8 ± 0.4	Noticeable improvement in right quadrants; uneven results in left quadrants. Mild discomfort but willingness to continue treatment.
After the second procedure	4.7 ± 0.3	High satisfaction due to uniform improvement across all quadrants, significant skin texture improvement, and reduced hyperpigmentation. Results exceeded expectations.

## Discussion

Sclerotherapy is a widely utilized, minimally invasive technique for the treatment of various venous disorders, including telangiectasias, reticular veins, and larger varicose veins, owing to its effectiveness, affordability, and ease of application. Despite its benefits, post-procedural complications such as hyperpigmentation remain a common concern. A systematic review by Bossart et al. reported hyperpigmentation rates ranging from 7.4% to 32.5% following polidocanol-based sclerotherapy for telangiectasias and reticular veins, with persistent pigmentation beyond one year observed in up to 17.5% of cases treated with foam sclerosants [1].

Nicolau syndrome, also known as embolia cutis medicamentosa, is a rare but severe complication following injections, leading to vascular occlusion, ischemia, necrosis, and subsequent skin damage. Several mechanisms contribute to the development of persistent hyperpigmentation in affected areas [2]. First, accidental arterial injection can lead to vascular obstruction and tissue necrosis, followed by post-inflammatory hyperpigmentation (PIH) due to increased melanin synthesis during healing. Second, vascular injury often results in red blood cell extravasation and iron breakdown, leading to hemosiderin deposition and a brownish discoloration. Additionally, the inflammatory response triggers melanocyte activation, further enhancing melanin production, particularly in individuals with darker skin types. In more severe cases, dermal remodeling and fibrosis can also occur, altering the skin’s texture and contributing to long-term pigmentation changes [3,4].

Various therapeutic strategies have been proposed in the literature to manage hyperpigmentation, particularly following Nicolau syndrome. Topical depigmenting agents and corticosteroids often have limited efficacy, especially when hemosiderin is involved [5]. Recent studies have explored the use of laser modalities such as Q-switched ruby lasers, pulsed dye lasers, and combinations of IPL with radiofrequency or other lasers to address complex pigmentary changes [6,7]. In particular, the combination of IPL, Q-switched Nd:YAG, and Er:YAG lasers has demonstrated promising outcomes in targeting melanin and hemosiderin through selective photothermolysis and fractional resurfacing [8].

In our case, a 40-year-old woman with Fitzpatrick skin type II presented with a 10-month history of persistent hyperpigmentation in the right popliteal area following polidocanol sclerotherapy. Clinical and dermoscopic evaluations revealed mixed pigmentary deposition consistent with both melanin and hemosiderin. The patient underwent two sessions of laser treatment using a quadrant-specific protocol combining IPL, Q-switched Nd:YAG, and Er:YAG lasers. Results showed significant pigment clearance, with IPL and Er:YAG combination therapy yielding the most favorable outcomes. These findings are consistent with previous literature demonstrating the superior efficacy of multimodal laser therapy compared to monotherapy in the treatment of pigmentary disorders. Several studies have shown that combining different laser modalities targeting various chromophores and skin depths enhances pigment clearance, reduces treatment sessions, and minimizes recurrence rates. For example, the combination of IPL and Q-switched Nd:YAG lasers has been shown to provide greater improvement in epidermal and dermal pigmentation compared to either modality alone. Similarly, adding fractional Er:YAG to pigment-targeting lasers enhances epidermal remodeling and overall aesthetic outcomes [9-11].

Follow-up over several weeks demonstrated progressive pigment resolution, with complete crust exfoliation and restoration of normal skin tone in the treated quadrants. Independent expert evaluation confirmed marked clinical improvement, while the patient reported high satisfaction, particularly following the second session. These observations align with previous literature, which emphasizes the importance of individualized treatment protocols and regular follow-up in achieving optimal outcomes in pigmentary disorders [12]. Sustained pigment reduction over time, as seen in our case, reinforces the value of multimodal laser therapy as an effective, safe, and well-tolerated strategy for treating complex post-inflammatory hyperpigmentation, particularly when guided by objective assessments and patient-reported outcomes.

## Conclusions

This case highlights the potential of combining IPL, Q-switched Nd:YAG, and Er:YAG lasers for the effective treatment of post-sclerotherapy hyperpigmentation secondary to Nicolau syndrome. By tailoring laser settings to address both melanin and hemosiderin across different skin depths, this multimodal approach offers a significant advantage over conventional therapies. The patient's favorable response, corroborated by clinical assessment and subjective satisfaction, underscores the clinical relevance of individualized, quadrant-specific treatment strategies. Furthermore, the absence of adverse effects and sustained pigment clearance during follow-up strengthens the argument for considering this protocol in similar clinical contexts. Continued research and broader clinical validation are warranted to establish standardized guidelines for the management of complex pigmentary complications with laser therapy.
